# The expression in plants of an engineered VP2 protein of Infectious Bursal Disease Virus induces formation of structurally heterogeneous particles that protect from a very virulent viral strain

**DOI:** 10.1371/journal.pone.0247134

**Published:** 2021-02-16

**Authors:** Carla Marusic, Charifa Drissi Touzani, Alessio Bortolami, Marcello Donini, Claudia Zanardello, Chiara Lico, Emile Rage, Siham Fellahi, Mohammed El Houadfi, Calogero Terregino, Selene Baschieri

**Affiliations:** 1 Laboratory of Biotechnology, ENEA Casaccia Research Center, Rome, Italy; 2 Avian Pathology Unit, Pathology and Veterinary Public Health Department, Agronomy and Veterinary Institute Hassan II, Rabat, Morocco; 3 Specialized Virology and Experimental Research Department Istituto Zooprofilattico Sperimentale delle Venezie, Legnaro, Italy; 4 Diagnostic Services, Histopathology, Parasitology Department, Istituto Zooprofilattico Sperimentale delle Venezie, Legnaro, Italy; Instituto Butantan, BRAZIL

## Abstract

Infectious Bursal Disease Virus (IBDV), the etiological agent of Gumboro disease, causes mortality and immunosuppression in chickens and major losses to poultry industry worldwide. The IBDV major capsid protein VP2 is considered the best candidate for the production of novel subunit vaccines. This structural protein contains the major conformational epitopes responsible for the induction of IBDV neutralizing antibodies in chickens and has been demonstrated able to form supramolecular structures in yeast and insect cells. The aim of this study was to express an engineered version of the VP2 protein (His-pVP2) to verify its ability to self-assemble into virus-like particles in plants. The recombinant VP2 was transiently expressed by agroinfiltration in *Nicotiana benthamiana* and transmission electron microscopy of sucrose density gradient fractions revealed the presence of a mixed population of differently shaped particles ranging from spherical capsids, with a diameter between ~25 and ~70 nm, to tubular structures, with variable length (from 100 to 400 nm). The recombinant VP2-based particles when used for the intramuscular immunization of specific-pathogen-free chicks resulted able to induce the production of anti-IBDV specific antibodies at titers comparable to those induced by a commercial vaccine. Moreover, all the immunized birds survived to the challenge with a Moroccan very virulent IBDV strain with no major histomorphological alterations of the Bursa of Fabricius, similarly to what obtained with the commercial inactivated vaccine.

## Introduction

The control of immunosuppressive diseases of poultry remains a major concern for farmers. Among these, Gumboro disease, caused by Infectious Bursal Disease Virus (IBDV), has a major economic impact on poultry farms worldwide [1]. Two serotypes of IBDV have been identified [2]: serotype 1, typically infecting chickens and causing immunosuppression and serotype 2 infecting a wide range of avian species, including turkeys, without causing evident symptoms. The viruses belonging to serotype 1 have been classified into sub-clinical (scIBDV), classic virulent (cvIBDV) and very virulent (vvIBDV) strains on the basis of the severity of the disease they induce [3]. After oral infection or inhalation, the virus starts to replicate in the lymphocytes and macrophages of gut-associated lymphoid tissues and then, through the blood stream, migrates to the Bursa of Fabricius (BF) an avian primary lymphoid organ [4]. Here the virus induces the progressive loss of immature B lymphocytes and this determines manifestations that may range from increased susceptibility to opportunistic infections to death [4]. The vvIBDV, identified for the first time in Belgium during the early 1980s, is the most aggressive towards B lymphocytes and for this reason produces a severe immunosuppressive disease associated to high mortality [5, 6]. Phylogenetic analysis indicates that this viral pathotype has spread worldwide causing considerable economic losses [1]. For this reason, the development of safe and low-cost vaccines is mandatory and extremely urgent [7, 8]. Currently, most of the available vaccines consist of inactivated or live attenuated viruses. The last ones mimic the viral infection by replicating in the host and inducing both cellular and humoral immunity but have proved to be not totally effective in inducing protection from vvIBDV strains. Moreover, even if suitable for mass administration to chickens, they may have undesirable effects due to a possible risk of reversion to virulence or to adverse vaccine reactions that may evolve in animal sickness and death. To avoid unwanted secondary effects, a second generation of vaccines have been developed adopting new technologies ranging from genetically engineered viruses to recombinant subunit vaccines [7].

IBDV is an icosahedral virus belonging to the *Birnaviridae* family, genus *Avibirnavirus* and its capsid (60–70 nm in diameter) organized in a T = 13 icosahedral lattice consists of a single shell formed by 260 trimers of the main coat protein (VP2), a variable amount of the VP2 precursor (pVP2) and the VP3 protein [9–12]. Structural studies have demonstrated the ability of different VP2 deletion mutants to self-assemble into supramolecular structures with quasi-equivalent icosahedral symmetries (from T = 1 sub-viral particles, SVP, to T = 13 virus-like particles, VLP) [12–14]. The self-assembly ability of the VP2 combined with the capacity to induce the production of neutralizing antibodies in chickens for the presence of major conformational epitopes [15] makes it the best candidate for the formulation of novel recombinant IBDV subunit vaccines. With this goal, VP2-based structures have been obtained by expressing the protein in yeast [16, 17] and insect cells [13, 18]. Some studies have also shown the possibility to successfully produce the VP2 in different plant species (*Nicotiana tabacum*, *N*. *benthamiana*, *Oryza sativa* and *Arabidopsis thaliana*) [19]. The plant-produced monomeric VP2 was able to elicit protective IBDV-specific humoral responses in chickens [20–22]. Recently a paper was published showing also the assembly in plants of the VP2 in SVP and the use of these structures to develop a diagnostic tool [23]. Nonetheless, the ability of plant-produced VP2-based structures to induce protection against IBDV infection has never been demonstrated [20, 24–26].

In this study, the engineered version of the pVP2 N-terminally fused to His tag (His-pVP2) was transiently expressed in *N*. *benthamiana*. Transmission electron microscopy (TEM) of leaf extracts was performed to verify the ability of the recombinant protein to self-assemble into supramolecular structures. Moreover, the immunization and challenge of specific-pathogen-free (SPF) chicks was reported.

## Material and methods

### Cloning

**Fig 1:**
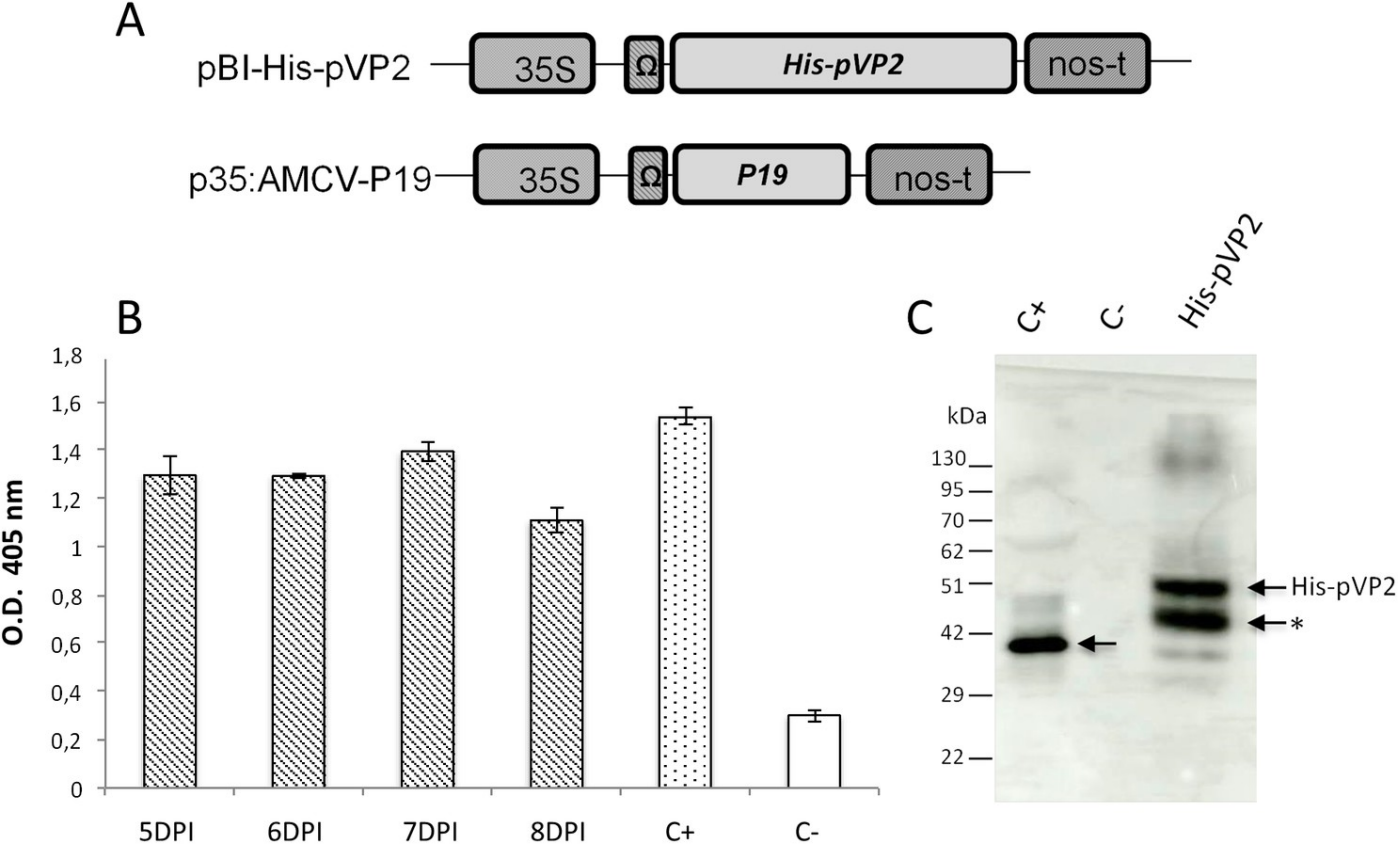
Expression of recombinant VP2 in *N*. *benthamiana*. A) Schematic representation of pBI-His-pVP2 and p35:AMCV-P19 constructs used for plant agroinfiltration. 35S: CaMV 35S promoter; Ω: TMV translational enhancer sequence; *His-pVP2*: sequence encoding the first 466 aa of the pVP2 protein N-terminally fused to His-tag; *P19*: sequence encoding the AMCV p19 gene-silencing suppressor; nos-t: *nopaline synthase* gene terminator. B) Evaluation by ELISA of His-pVP2 expression in agroinfiltrated leaf extracts at different time points (5, 6, 7, 8 DPI). Fifty μg of TSP extracted from agroinfiltrated leaves were distributed in triplicate into the wells and His-pVP2 was detected with an anti-VP2 antibody. The reported values are the mean of three independent experiments, and error bars represent SD of the means. C+: inactivated IBDV; C-: p19 plant extract. C) Western blot analysis of His-pVP2 expression in leaves extract at 7 DPI. Ten μg of TSP were separated by 11% SDS-PAGE and His-pVP2 was detected using an anti-VP2 serum. C+: inactivated IBDV; C-: P19 agroinfiltrated leaf extract. His-pVP2: His-pVP2 agronfiltrated leaf extract. The arrow indicates the band corresponding to His-pVP2 while the asterisk a possible degradation product.

The *His-pVP2* synthetic gene (GenBank Accession Number MT780551) was obtained by fusing the nucleotide sequence encoding a poly-histidine tag (His) [13] to the 5’-end of the VP2 encoding sequence (*pVP2*) constructed by fusing the sequence encoding amino acids 1–452 of the IBDV VP2 protein (GenBank Accession Number JN982254) to that encoding amino acids 453–466 of the IBDV polyprotein (GenBank Accession Number AF140705.1). The nucleotide sequence of the synthetic gene (Eurofins Genomics, Ebersberg, Germany) was optimised for the expression in *N*. *benthamiana* using the GENEius software (Eurofins Genomics, Ebersberg, Germany) and cloned in the pBI-Ω plant expression vector [27] under the control of the Cauliflower Mosaic Virus 35S promoter (35S), the TMV translational enhancer sequence (Ω) and the *Agrobacterium tumefaciens* (*A*. *tumefaciens*) nopaline synthase gene terminator (nos-t) using the *Bam*HI/*Eco*RI restriction sites, yielding the pBI-His-pVP2 construct (Fig 1A). The p35:AMCV-P19 construct encoding for the Artichoke Mottled Crinkle virus (AMCV) P19 silencing suppressor protein was previously described [28].

### Plant agroinfiltration and His-pVP2 transient expression

*N*. *benthamiana* plants (NCBI:txid4100) were grown in a greenhouse at 24°C under controlled conditions of light (16/8 h light/dark cycle). Transient expression of the recombinant pVP2 was obtained by vacuum infiltrating the plants at the 6–7 leaf stage with suspensions of LBA4404 *A*. *tumefaciens* (Thermo Fisher Scientific, Rockford, IL, USA) harbouring the pBI-His-pVP2 plasmid or the p35:AMCV-P19 construct. The two bacterial strains were grown separately in LB medium overnight at 28°C. After centrifugation at 4000 xg, the cells were resuspended in the infiltration buffer (10 mM MES, 10 mM MgCl_2_, pH 5.8) and then mixed together to reach the optical density (OD_600_) of 0.4 for each clone. As negative control (C-) some plants were infiltrated only with *Agrobacterium* harbouring p35:AMCV-P19. The infiltration was performed in a vacuum chamber applying a pressure of ~10 mm Hg. The plants were then left in the greenhouse and leaf samples harvested at different time points (5, 6, 7 and 8 days post infiltration, DPI) to determine maximum His-pVP2-accumulation levels. Thereafter, to perform particles purification the leaves were collected at 7 DPI, divided into batches of 20 g, frozen in liquid N_2_ and stored at -80°C.

### ELISA and Western blot analysis

The plant tissues (200 mg) (collected at 5, 6, 7, 8 DPI) were ground in liquid N_2_ and homogenised by disposable pestles in 400 μl of phosphate-buffered saline (PBS) pH 7.2 containing a protease inhibitor cocktail (cOmplete^TM^; Roche, Mannheim, Germany). Crude plant extracts were clarified by centrifugation at 20000 xg for 20 min and the Total Soluble Protein (TSP) content was determined by Bradford colorimetric assay following the manufacturer’s protocol (Bio-Rad Protein Assay, Hercules, CA, USA). To perform Enzyme Linked ImmunoSorbent Assay (ELISA), 100 μl (50 μg TSP) of each supernatant were distributed in triplicate into the wells of Nunc-Immuno Maxisorp plates at 4°C overnight. After washes, the wells were incubated with 2% (w/v) milk in PBS at 37°C, for 2 h. Then a rabbit anti-VP2 serum (kindly provided by Prof. J.R. Castón, Centro Nacional de Biotecnología/CSIC, Cantoblanco, Madrid, Spain) diluted 1:1000 in 2% milk-PBS was added and incubated overnight at 4°C [29]. After washing, the detection was performed with a goat anti-rabbit Horse Radish Peroxidase (HRP)-conjugated antibody (Goat anti-Rabbit IgG (H+L) Secondary Antibody, HRP, ThermoFisher Scientific, Rockford USA) diluted 1:5000 in 2% milk-PBS for 1 h at 37°C. Enzymatic activity was measured at 405 nm by a microtiter plate reader (TECAN-Sunrise, Groedig, Austria) adding to the wells 2,2-azino-di-3-ethylbenz- thiazoline sulphonate (ABTS, KPL). As positive and negative controls, inactivated IBDV (provided by IZSVe) and the extract of plants infiltrated only with p35:AMCV-P19 were used respectively.

ELISA of sucrose gradient density fractions was performed by coating triplicate wells with 100 μl of each fraction.

Quantitative ELISA of VP2-derived particles, purified on sucrose cushion, was performed by coating in triplicate the wells with serial dilutions of the plant-purified particles and serial dilutions of purified IBDV (gently provided by IZSVe) starting from a stock at the concentration of 40 ng/μl. In both cases the following ELISA steps were performed exactly as previously described using a rabbit anti-VP2 serum as primary antibody.

For Western blot analysis of plant agroinfiltrated leaves, 10 μg of extracted TSP were separated by 11% SDS-PAGE, before electro-transfer to a polyvinylidene fluoride (PVDF) membrane (Millipore, Bedford, MA) using a Semi-Dry Transfer Unit (Hoefer TE70; GE Healthcare, Freiburg, Germany). As positive and negative controls, inactivated IBDV (provided by IZSVe) and the extract of plants infiltrated only with p35:AMCV-P19 were used respectively. The virus used as positive control (C+), was inactivated with 0.4% formaldehyde for 24 hours. The membranes were then blocked with 4% milk-PBS 2 h at room temperature (r.t.), and after washing, the rabbit anti-VP2 serum diluted 1:1000 in 2% milk-PBS was added and incubated overnight at 4°C. After washings, the goat anti-rabbit HRP-conjugated antibody (ThermoFisher Scientific, Rockford USA) diluted 1:5000 in 2% milk-PBS for 1 h at 37°C was added. Proteins were detected by enhanced chemiluminescence (ECL, Plus; GE Healthcare) using an ImageQuant^TM^ LAS 500 system (GE Healthcare, Uppsala, Sweden).

### Purification of VP2-based particles

His-pVP2 agroinfiltrated plant tissues (20 g) were ground in liquid N_2_ and then homogenised with Ultraturrax T25 (IKA-Werke, Staufen, Germany) in 60 ml of a buffer containing 25 mM piperazine N,N′-bis(2-ethanesulfonic acid) pH 6.2, 150 mM NaCl, 20 mM CaCl_2_, 1% cOmplete^TM^ (Roche, Mannheim, Germany) (PES buffer) [13]. After incubation on ice for 30 min, the extract was filtered through Miracloth (Sigma-Aldrich), clarified by centrifugation at 12000 xg, 4°C for 15 min and the supernatant layered on a 20% sucrose cushion that was centrifuged at 170000 xg, 4°C, 150 min. The pellet was resuspended in 1 ml of PES buffer final volume, layered on a 20–50% linear sucrose gradient and centrifuged again at 200000 xg, 4°C, 45 min. Twelve fractions of 1 ml were collected, concentrated using Amicon^®^ Ultra-4 Centrifugal Filter Devices (Merck KGaA, Darmstadt, Germany) and analysed by Western blot as described above. For the immunization experiments VP2-derived particles purified by processing the plant extract only on the 20% sucrose cushion were used.

### Transmission electron microscopy

For negative staining, samples were fixed using 4% paraformaldehyde (PFA) in PBS. Droplets of sample suspensions (10 μl) were placed on formvar-carbon coated copper grids and allowed to adsorb for 60 sec. Excess liquid was removed gently touching the filter paper. The adsorbed specimens were then processed by first washing each specimen grid on a drop of negative stain (2% uranyl acetate in distilled water), blotting and repeating this step once more, this time leaving the specimen grid for 60 sec on a new drop of negative stain solution. Samples were observed at a JEOL 1200 EX II electron microscope. Micrographs were acquired by the Olympus SIS VELETA CCD camera equipped the iTEM software.

### Animal immunization

Twenty-one SPF chicks of 8 days of age (kindly provided by MCI Animal Health Laboratories, Mohammedia, Morocco) were divided into three groups of six birds and injected intramuscularly with a preparation of VP2-based particles (VP2-BP), PBS (negative control, C- group) or the commercial IBD vaccine containing inactivated Gumboro virus strain D78 in oil adjuvant emulsion. (NOBILIS® GUMBORO inac; https://www.msd-animal-health-maghreb.com/produits/nobilis-gumboro-inac/) (positive control, C+ group). In details, the chickens of the first and second group were immunized with 35 μg of VP2-based particles or with PBS, emulsified in a 2:1 ratio of oil adjuvant (Montanide^TM^ ISA 71, kindly provided by Biopharma Laboratories, Rabat, Morocco). All the birds were injected at day 8, 21 and 35 post-hatch (D8, D21, D35). Three SPF chickens were left untreated (healthy chickens) and used as a control for the histopathological studies.

All animals were kept with a 12 hours daylight period with unrestricted access to ad libitum feed and water. No analgesics or anaesthetics were used in the experimental procedures to avoid interference with the recording of clinical data. The total duration of experiments was 45 days.

All the animal experiments were carried out in accordance with European and French legislations on care and use of laboratory animals (French Decree 2001–464 and European Directive CEE86/609) following animal protocols approved by the Ethics Committee ‘‘Sciences et santé animale”, committee number 115. Chickens were kept in the animal facilities (biosafety level 2) of the Agronomy and Veterinary Institute Hassan II, Rabat Morocco.

All research personnel involved in animal studies received training in the ethical care and use of animals.

### Serum antibody titration

Blood samples (2–3 ml) were collected one day before each immunization and the day before the challenge (D7, D20, D34, D42) and stored at -20°C. Anti-IBDV antibody titers were determined using a commercial kit (ProFLOCK^®^ infectious bursal disease virus antibody test kit, Synbiotics Corporation, San Diego, USA), according to the manufacturer’s instructions. Briefly, 1:50 serum dilutions were obtained by adding 6 μl of serum into 300 μl of dilution buffer. Diluted sera were then added to a IBDV coated plate (50 μl of diluted serum + 50 μl of dilution buffer in each well), and incubated 30 min at r.t. After the wash procedure (300 μl washing buffer per well repeated three times), 100 μl of anti-chicken IgG peroxidase conjugated were added and incubated for 30 min. Then, 100 μl of substrate solution were added after wash procedure. The reaction was stopped by adding 100 μl of stop solution. All reagents were provided by the manufacturer.

### Virus challenge

Chickens of the VP2-BP, C+ and C- groups were infected at D43 (8 days after the last immunization) via the oculo-nasal route with 0.2 ml of a suspension titrated to 10^5^ EID50 (50% egg-infective dose) [30] of a vvIBDV strain (accession number: MF320263) isolated from broiler chickens in Morocco in 2017 [31]. Clinical signs and mortality were recorded daily during the following 10 days. A humane endpoint was determined when chickens showed severe prostration and inability to reach feed or water. All the animals were euthanized by cervical dislocation when the criteria set for the humane endpoint were met. All surviving chickens (VP2-BP, C+ and untreated groups) were euthanized 10 days after challenge by cervical dislocation. Before euthanasia, the chickens were anesthetised by injection with 1 ml of Dolethal (Dolethal, Vétoquinol, Cedex, France) to provide a peaceful and painless death. Post-mortem examination was performed on all animals (C-, C+, VP2-BP and Untreated groups).

### Preparation of tissues for histological study

The frozen bursae were placed in 10% neutral-buffered formalin and paraffin embedded. Three μm sections were cut, stained with hematoxylin and eosin (HE) and examined under light microscope. The lesions were scored using the method described by Muskett and colleagues [32]. In brief, score 0 was assigned to BF free of lesions. Score 1 to BF where lymphoid depletion and accumulation of heterophils was evident in 1% to 25% of the follicles but with less than 50% of depletion/follicle. Score 2 was assigned when 26% to 50% of the follicles show more than 75% depletion of lymphoid cells and accumulation of heterophils was accompanied by signs of necrosis. In score 3 organs almost complete lymphoid depletion was evident in 51% to 75% of the follicles with necrosis and heterophils. This percentage was raised to 76% to 100% in score 4 organs where necrosis, heterophils, hyperplasia and cysts were also observed and to 100% in score 5 organs that were fibrous and lost the typical bursa architecture.

### Viral RNA quantification in bursal tissue

Bursal tissues (300 mg) were homogenized with mortar, pestle and sterile quartz sand, and diluted at 1:10 (w/v) with PBS containing antibiotics and antimycotics (1X Antibiotic Antimycotic Solution, Sigma-Aldrich, USA). Nucleic acids were isolated from 300 μl of tissue homogenate with the QIAsymphony^®^ DSP Virus/Pathogen Midi Kit (Qiagen GmbH, Germany) on a QIAsymphony^®^ SP instrument using a custom protocol provided by Qiagen.

IBDV was subject to absolute quantification by real-time reverse transcription polymerase chain reaction (qRT-PCR) targeting the VP4 gene [33] with the QuantiTect Multiplex RT-PCR Kit (Qiagen). Briefly, each reaction contained: 12.5 μl of 2x QuantiTect Multiplex RT-PCR Master Mix, 250 nM of each primer, 300 nM of IBDV-Very-Virulent probe (FAM– 5′-CAACGCCTATGGCGAGATTGAGAACGTGAG-3′– TAMRA), 0.25 μl of QuantiTect® Multiplex RT Mix, 5 μl of template RNA and RNase-free water up to 25 μl. The qRT-PCR were run on a Rotorgene 6000 (Qiagen), under the cycling conditions herein reported: 50°C for 20 min and 95°C for 15 min, followed by 40 cycles at 94°C for 45 sec and 60°C for 45 sec. Each sample was tested in triplicate.

For absolute virus quantification, a standard curve was taken along with the samples. In detail, log_10_ serial dilutions of vvIBDV isolate (L1/08) previously titrated *in vivo* (10^6.375^ Chicken Infectious Dose 50, CID_50_/100μl) were subject to RNA isolation as described above. Each dilution of purified RNA was tested in triplicate.

Amplification data were analyzed with the Rotorgene Q series software (Qiagen®). The standard curve was used to determine amplification efficiency and to convert mean Ct values of unknown samples into CID_50_/100μl equivalent by interpolation. Viral load in the BF samples were plotted using GraphPad Prism software version 8.3.0.

### Statistical analysis

Statistical analysis was performed using GraphPad Prism for Windows, GraphPad Software, La Jolla, CA, USA, www.graphpad.com. Student’s t-test on. *p-*values < 0.05 were considered statistically significant.

## Results

### Cloning and expression of His-pVP2 in *N*. *benthamiana* plants

The *His-pVP2* gene encoding the first 466 amino acid residues of pVP2 fused at the N-terminus to a poly-histidine tag (His) was cloned in the pBI-Ω plant expression vector (Fig 1A). *N*. *benthamiana* plants were infiltrated with a mix of *A*. *tumefaciens* strains carrying pBI-His-pVP2 and p35:AMCV-P19 constructs (Fig 1A). As negative control, a group of plants was infiltrated only with *Agrobacterium* bearing p35:AMCV-P19.

Agroinfiltrated leaves were harvested at 5, 6, 7 and 8 DPI and extracts normalized for TSP content were used to coat the wells of ELISA plates in order to verify the expression of the His-pVP2 protein (Fig 1B). Results showed that the highest accumulation level was reached at 7 DPI. The analysis by SDS-PAGE followed by Western blot (Fig 1C), revealed the presence of two major bands, one migrating with an apparent mass of ~ 48 kDa, corresponding to the expected size of the VP2 recombinant protein, and one with an apparent mass of ~ 45 kDa (indicated with an asterisk) probably corresponding to a degradation product. An additional fainter band of ~ 130 kDa was also visible possibly indicating the presence of high molecular weight aggregates. A major band of ~ 41 kDa corresponding to the wild-type VP2 was visible in purified inactivated IBDV (C+).

### Purification and characterisation of VP2-based structures

**Fig 2:**
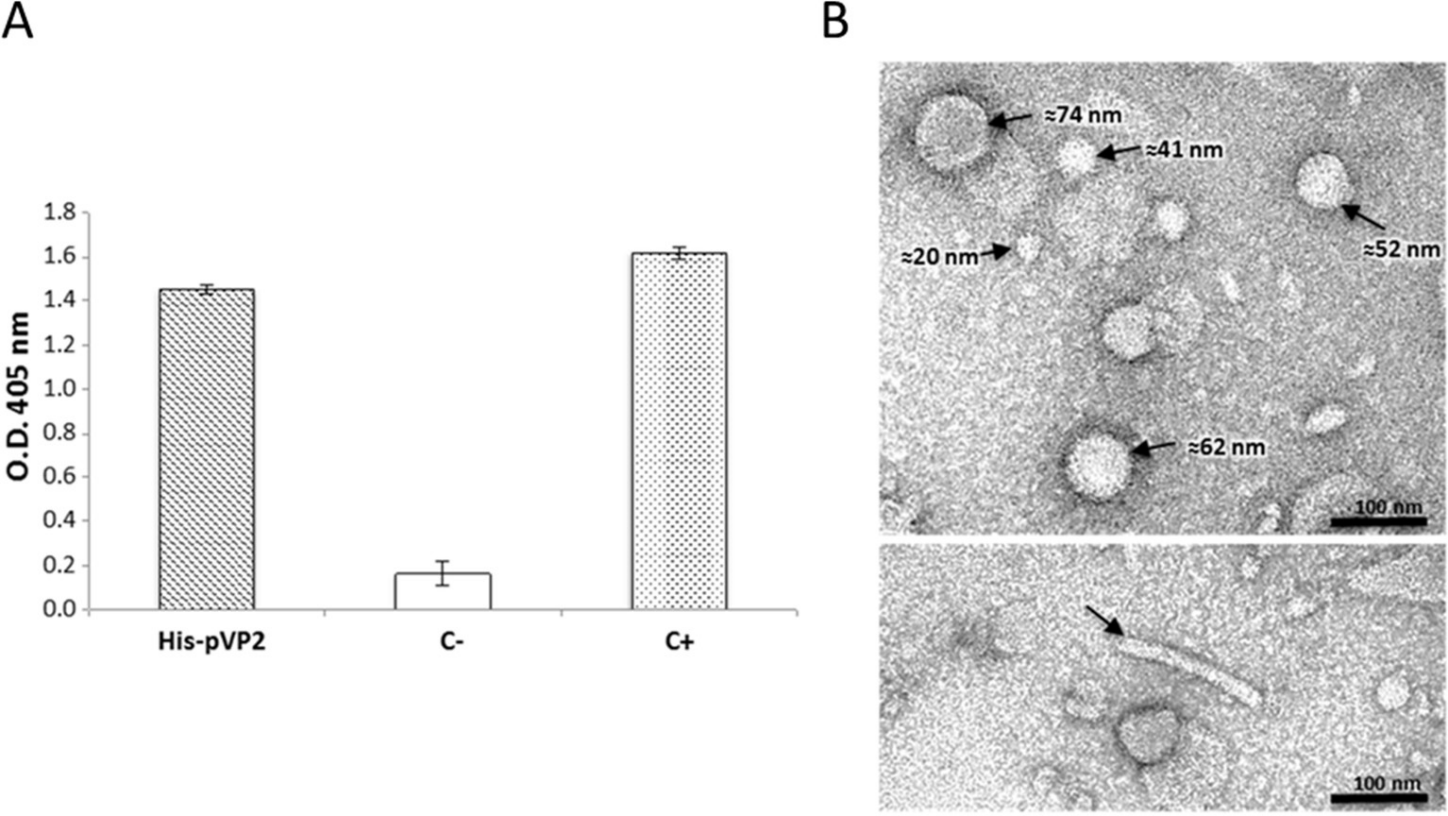
Assembly of His-pVP2 into supramolecular structures in plant tissues. A) ELISA with an anti-VP2 serum of the sucrose cushion resuspended pellets. His-pVP2: pellet obtained with the His-pVP2 extract; C-: pellet obtained with the p19 extract; C+: inactivated IBDV. The reported values are the mean of three independent experiments, and error bars represent SD of the means. B) TEM images of the plant-produced VP2-based particles. In the upper panel, the different spherical particles and their measured diameters are indicated. The example of a tubular structure is shown in the lower panel.

To verify the ability of His-pVP2 to self-assemble into supramolecular structures in plant tissues, the 7 DPI leaf extract was loaded on a 20% sucrose cushion, purified by ultracentrifugation, and the pellet analysed by ELISA using an anti-VP2 antibody (Fig 2A). A strong signal was detected in the wells coated with the resuspended pellet, indicating the ability of His-pVP2 to form high-density supramolecular assemblies. The morphology and dimensions of these assemblies were defined by TEM on negatively stained grids. The images clearly showed that His-pVP2 was self-assembled into spherical SVP and VLP with diameter ranging from ~25 to ~70 nm (Fig 2B upper panel) but also into tubular structures with diameter of ~22 nm and length in the range of 100–400 nm (Fig 2B lower panel).

**Fig 3:**
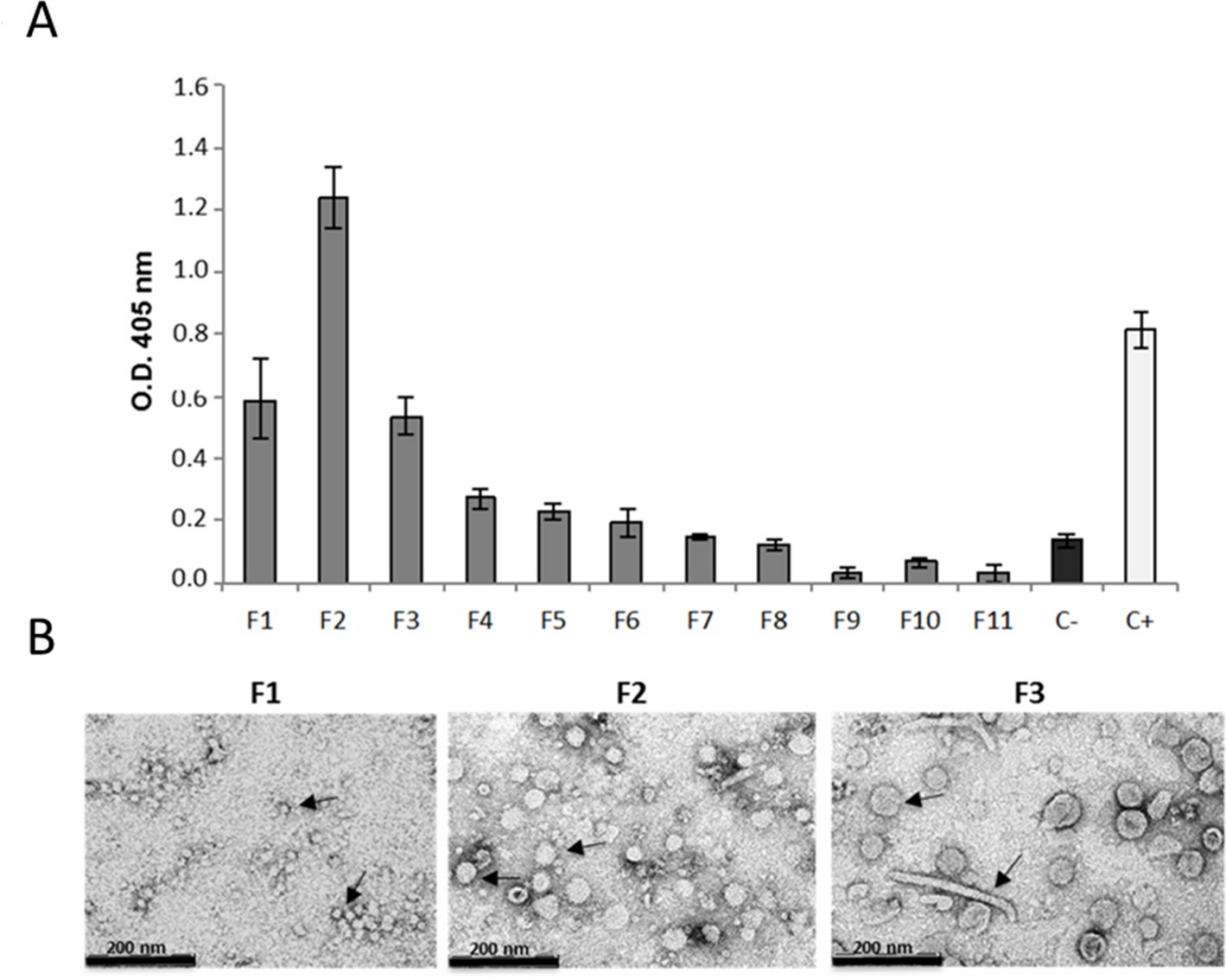
Separation of the His-pVP2 particles by sucrose gradient. a) ELISA with an anti-VP2 antibody of the sucrose gradient fractions. The fractions were numbered from 1 (F1; top of the tube) to 11 (F11; bottom of the tube). C-: p19 plant extract; C+: inactivated IBDV. b) TEM images of sucrose gradient fractions. In F1 only SVP (T = 1) are present, indicated by the arrows. F2 contains bigger particles with a diameter ranging from 35 to 50 nm (T = 7). In F3 the majority of the particles are VLP with a diameter of ~ 60 nm, (T = 13), but tubular structures are also visible.

The resuspended sucrose cushion pellet was further separated through a sucrose gradient and fractions were collected from the top (fraction 1, F1) to the bottom (fraction 11, F11) of the tube. The ELISA of the different samples (F1 to F11) performed using the anti-VP2 antibody showed a strong signal in the first three fractions of the gradient with the highest amount of His-pVP2 in F2 (Fig 3A). The TEM images of these fractions showed the presence in F1 of very small particles with diameters ranging from ~15 nm to ~20 nm, probably corresponding to T = 1 shells (SVP) (Fig 3B, left panel). Sample F2 was shown to contain mainly particles with size in the range of ~35–50 nm possibly corresponding to T = 7 capsids (Fig 3B, middle panel), while in F3, the majority of the observed particles had a diameter of about 60 nm, corresponding to the expected size of VLP (T = 13). In this fraction, tubular structures were also present (Fig 3B, right panel).

The overall yield of VP2-based particles, estimated by quantitative ELISA was of 12.5 mg/kg of leaf fresh weight.

### Immunization experiments and evaluation of the antibody response

**Fig 4:**
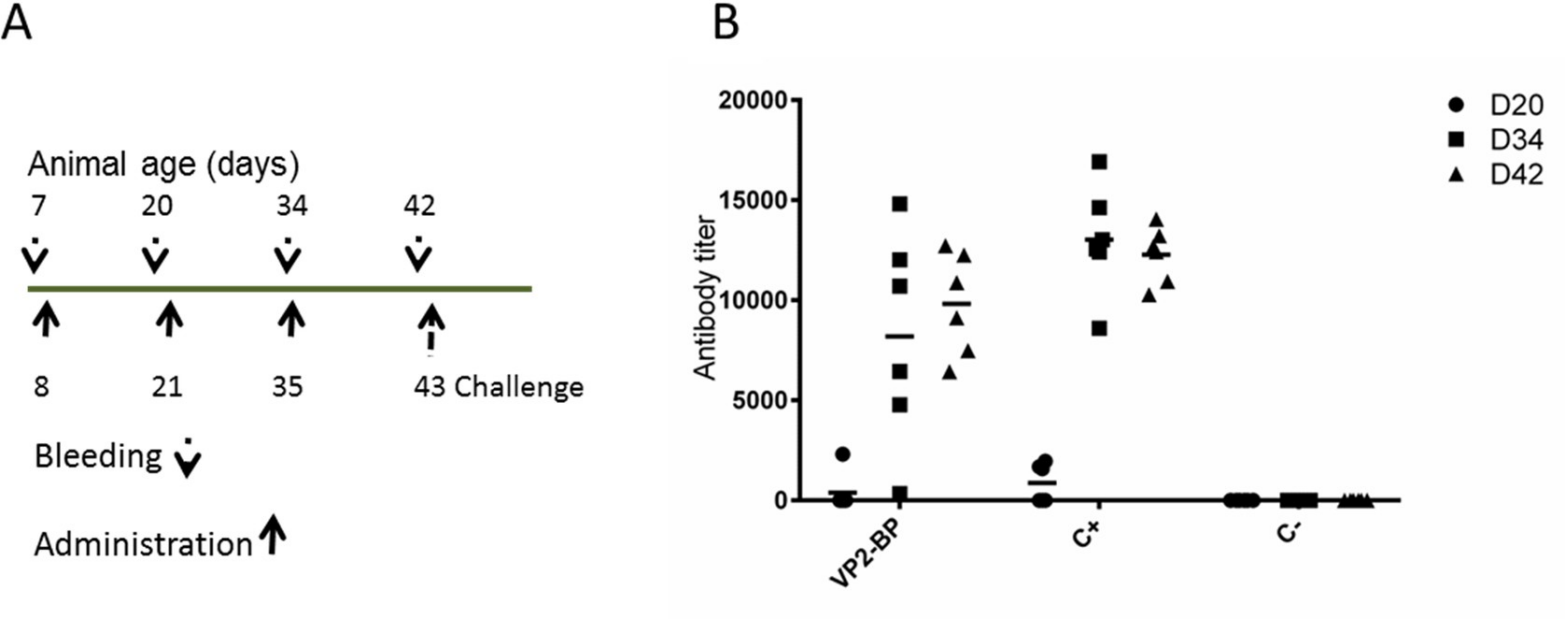
Chickens immunization and antibody response. A) SPF chickens immunization schedule. B) Anti-VP2 antibody titers in the sera of the immunized animals. (VP2-BP) sera of the chickens injected with the VP2-based particles; (C+) sera of the chickens injected with the inactivated commercial vaccine; (C-) sera of PBS injected animals. Individual antibody levels at different time points are reported (D20, 34 and 42). The horizontal lines represent the average antibody titers in each group at different time points. Antibody titers values in the sera of all animals at D34 and D42 in VP2-BP and C+ are significantly higher compared to C- (Student’s *t*-test *p* < 0.05).

The assembled particles obtained by passing the plant extract through the sucrose cushion (mainly spherical capsids of different diameters as well as tubular structures) (Fig 2B and 2C) were injected through the intramuscular route in SPF chicks at day 8 (D8), D21 and D35 post-hatching (Fig 4A). Sera were collected from each bird one day before each immunization and before the challenge (D7, D20, D34, D42) (Fig 4A), and the presence of VP2-specific antibodies evaluated by ELISA (Fig 4B). Before the second immunization (D20), antibodies started to be detectable in one bird out of six in the group injected with the VP2-based particles (VP2-BP) with an antibody titer of ~2300, and in three birds out of six in the vaccine-injected group (C+) with antibody titers ranging between ~ 1600 and ~1900. At day 34, one day before the third immunization, five birds out of six were positive in the VP2-BP group with an average antibody titer of ~ 8000. All birds in the C+ group were positive showing average titers of ~ 13000. After the third immunization (D42) the antibody average titers in the sera of the VP2-BP immunized chickens (~10000) were comparable to those of the C+ group (~13000) (Fig 4B). As expected the sera of the C- group chickens (PBS injected) were negative at all time points.

### Post-challenge studies

At day 43 of age, all the animals were challenged *via* the oculo-nasal route with a titrated suspension of Moroccan vvIBDV strain (10^5^ EID50). All the chickens belonging to the C- group died within 5 days after the challenge while for VP2-BP or C+ animals no mortality was observed. Moreover, no symptoms of the disease, such as prostration, ruffled feathers or white watery diarrhoea, were observed in the animals belonging to these two groups 10 days after challenge when all the animals were sacrificed and BF collected for morphological and histopathological analyses.

**Fig 5:**
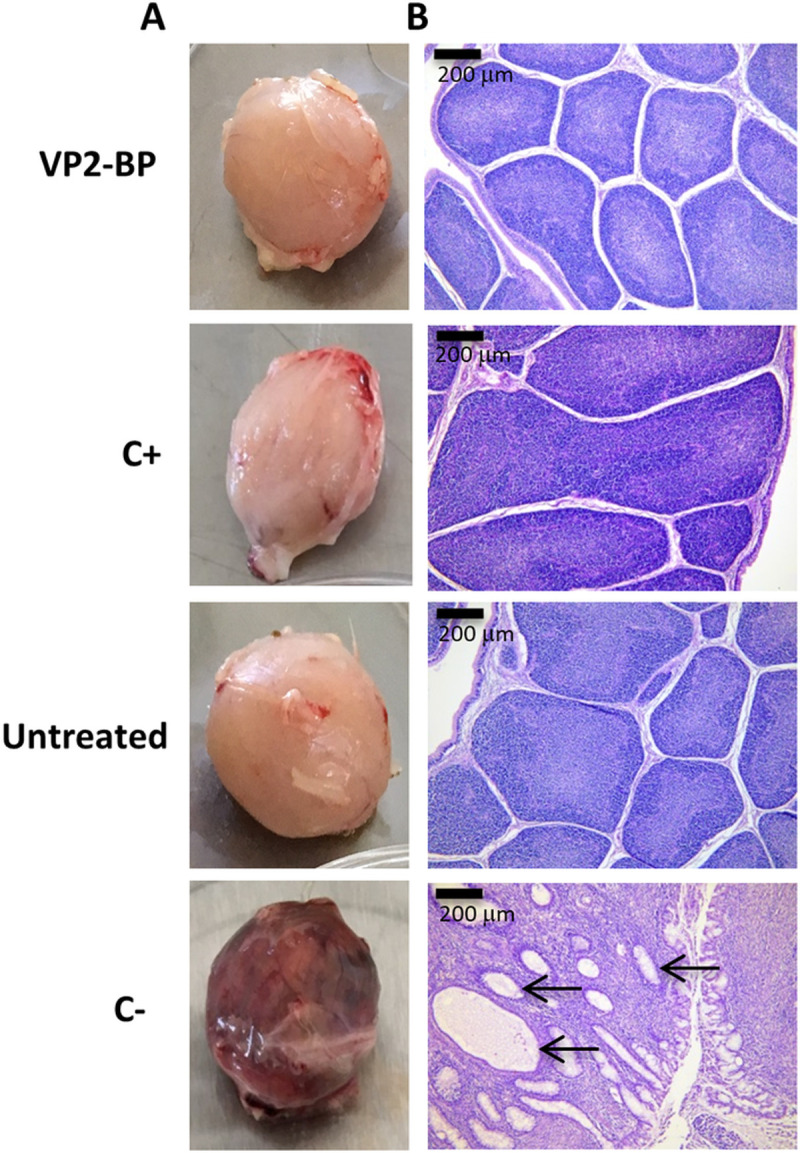
Macroscopic and histopathological analysis of BF. A) Representative images of the differences in the morphological aspect of BF collected 10 days post challenge in the different groups: (VP2-BP), vvIBDV challenged chickens immunized with the VP2 particles, showing normal appearance; (C+), vvIBDV challenged chickens immunized with the inactivated commercial vaccine, showing normal appearance; (Untreated), unchallenged naive chickens, showing normal appearance; (C-), vvIBDV challenged naive chickens, showing diffused hemorragic appearance. B) Histopathological pictures are representative of BF sections derived from 6 animals for each group and stained with hematoxylin and eosin. Diffuse necrosis of lymphocytes and marked atrophy of follicles are evident in the bursae of C- group. Loss of lymphocytes, within both medullary and cortex areas, results in multifocal, cystic cavities containing cellular debris (black arrows).

No morphological changes or significant macroscopic lesions were observed in the organs of the animals belonging to the VP2-BP and C+ groups which were comparable to those of the healthy untreated chickens. On the other hand, the BF of the C- group appeared grossly enlarged and haemorrhagic (Fig 5A).

Histopathological analysis of the BF of chickens of the VP2-BP group showed the absence of lesions (score 0) in 4 out of 6 animals, and score 2 and 4 lesions in the two remaining animals. The symptomatic organs were characterized by oedema, depletion of lymphoid cells in the follicles and infiltration with macrophages and heterophils. Histopathological lesions in the C+ group ranged from absent (score 0, 5/6 animals) to mild lymphoid cells depletion in less than 25% of the follicles (score 1, 1/6 animals). All the BF sections obtained from the animals of the C- group showed severe lymphoid cell depletion with necrosis, infiltration with heterophils and loss of the normal follicular architecture (mean lesion score 4.5) (Fig 5B). No lesions were observed in the untreated group (not vaccinated, not challenged).

**Fig 6:**
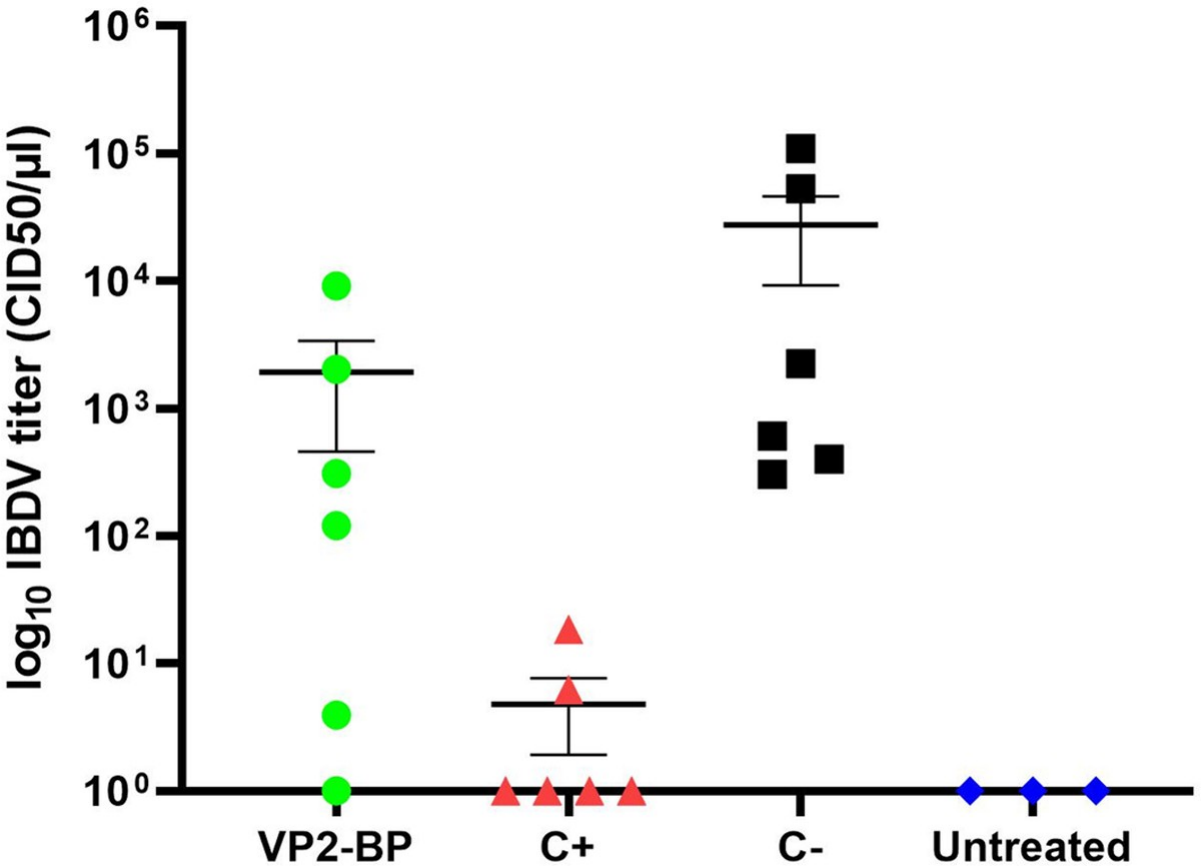
Quantification of IBDV load in bursal tissue of SPF chickens in the different treatment groups by qRT-PCR. Viral load in the BF of VP2-BP, C+ and C- groups was quantified 10 days post challenge by qRT-PCR. Chickens (six per group) were vaccinated with three doses of VP2-BP or the commercial IBD vaccine containing inactivated Gumboro (C+) or PBS as negative control (C-). Three untreated not-challenged birds served as control group (Untreated). IBDV RNA loads were determined using a standard curve and individual values expressed as log_10_ CID_50_/μl were plotted on the scatter plot shown. Horizontal lines indicate means with error bars.

The qRT-PCR analysis of bursal tissue homogenates showed presence of viral RNA in all challenged animals except for one belonging to the VP2-BP group (Table 1 and Fig 6). Statistically significant higher mean viral loads (Student’s t test, p < 0.05) were observed in the C- group (2.8x10^4^ CID_50_/μl) compared to the C+ group (4.3x10^0^ CID_50_/μl) (Fig 6). In the case of the VP2-BP group lower mean viral loads (1.9x10^3^ CID_50_/μl) were detected compared to the C- group although the difference was not statistically significant. The two animals of the VP2-BP group showing the highest viral loads (2x10^3^ CID_50_/μl and 9.1x10^3^ CID_50_/μl) (Fig 6) were those showing the highest BF lesion scores in the histopathological analysis (2 and 4, respectively).

**Table 1:** Effects of challenge with vvIBDV on immunized chickens.

Groups	Vaccine type	Number of dead birds/total	BF macroscopic lesions	Histopathological BF* mean lesion score	qRT-PCR positives
				± SD	(Average Ct)
VP2-BP	VP2-based particles	0/6	Normal	1.0± 1.67	5/6
					(30.37)
C+	GUMBORO inac	0/6	Normal	0.17± 0.41	6/6
					(35.72)
C-	PBS	6/6	Haemorrhagic and hypertrophic	4.5± 0.55	6/6
					(27.35)
Untreated	-	0/3	Normal	0	0/3
					(n.d.)

* The lesions were scored using the method described by Muskett *et al*., 1979 [32]. Student’s t test, p < 0.05 for VP2-BP and C+ versus C-.

BF: Bursa of Fabricius.

## Discussion

IBDV, the etiological agent of Gumboro disease, induces mortality and immunosuppression in young chickens causing major losses to poultry industry worldwide. The commercial vaccines available, mainly consisting of inactivated or live attenuated viruses, are not able to induce full protection against vvIBDV infection and may suffer from several biases as for example the possibility to revert to virulence. To overcome these limitations, a new generation of vaccines including subunit vaccines are under development [34, 35].

Among IBDV proteins, the VP2 that contains the major conformational neutralizing epitopes and is able to self-assemble into supramolecular structures (*i*.*e*. SVP, VLP and tubules) [18], is considered the best candidate to produce an effective recombinant subunit vaccine against Gumboro disease.

Until now VP2 in plants was mainly produced as monomer or trimer [26] and demonstrated to be fully effective as boost of the immune response to an attenuated commercial vaccine [22] while only partially efficient in conferring protection against viral challenge if used as a main immunogen [21, 24].

Although the assembly of the VP2 in capsid-like particles (mainly T = 1, T = 4 and T = 13 symmetries) or tubular structures in eukaryotic expression systems (yeast and insect cells) has been described [16–18], the formation of SVP in plants was only recently described and these structures used to set up a diagnostic assay [23].

In order to demonstrate the potentiality of plant-produced VP2 particles as protective vaccine, we transiently expressed in *N*. *benthamiana* an engineered version of the pVP2 derived from a vvIBDV field isolate, fused to a polyhistidine-tag (His-pVP2). Indeed, it was previously shown in a baculovirus-based expression system that this tag may mimic the C-terminal region of the VP3 which is critical for IBDV capsid assembly [13]. We showed that His-pVP2 was efficiently expressed in plant cells.

Interestingly, we demonstrated that the His-pVP2 variant assembles in plant cells not only into VLP (T = 13) but also into a range of different supramolecular structures such as SVP with a diameter ranging from ~15 nm to 20 nm (T = 1 shell), particles with a larger diameter (~35–50 nm), possibly corresponding to T = 7, and tubular structures of variable lengths (in the range of 100–400 nm) and ~22 nm in diameter. It is interesting that the formation of a comparable heterogeneous population of pVP2 supramolecular structures is observed in these two different systems (*i*.*e*. plant and insect cells), and this may indicate that similar protein assembly processes are implicated in both expression hosts.

Previous studies revealed that differently structured VP2-based particles are equally effective in inducing strong immune responses in chickens and in protecting animals from challenge with IBDV [18].

As the goal of the present work was to obtain a low-cost vaccine against IBDV by using a plant-based platform, the mixed population of particles simply and rapidly obtained from the crude plant extract through a sucrose cushion was used to immunise SPF chickens. Since no previous *in vivo* studies with plant-produced VP2-BP were available the adopted vaccination schedule was a minor modification of that used for a VP2 SVP yeast produced vaccine [17] and consisted in three intramuscular doses of 35 μg each. With this immunization scheme the antibody titers in the serum of chickens receiving the plant-produced particles (VP2-BP group) reached levels (~ 10000) comparable to those registered in the control group immunized with an inactivated commercial vaccine. These results are in line with the demonstration that high antibody titers were crucial for clinical protection in both experimental [36, 37] and field-settings [38]. Previous results obtained by immunizing three times with a plant extract containing the monomeric form of VP2 induced only low antibody titers (~ 4000) and as a consequence partial protection from challenge with a classical IBDV strain [24]. By the approach herein described it was also demonstrated for the first time that the strong anti-IBDV antibody response induced in chickens by the plant derived VP2-based particles was able to completely protect the animals from challenge with a vvIBDV strain and that 10 days after challenge all the animals were still alive. Moreover, the birds immunized with the VP2-BP did not show any symptoms of the disease, such as prostration or white watery diarrhoea, and morphological changes or macroscopic lesions of BF, differently from the C- group where the organ appeared enlarged and haemorrhagic. The histopathological analysis revealed a mean lesion score of 1 in the BF of the animals belonging to the VP2-BP group compared to a score of 0.17 in C+ and of 4.5 in the unvaccinated animals (C-).

The results of qRT-PCR analysis showed that even if immunization of birds with either the commercial inactivated vaccine or with the VP2-BP was able to protect the animals from clinical disease and mortality, the virus was still present, although at reduced levels, in the bursa of all birds except one (belonging to the VP2-BP group). The two birds of the VP2-BP group exhibiting higher viral loads comparable to those of the animals in the C- group were those showing the most evident histopathological BF lesions. The presence of the virus in vaccinated animals after challenge is not surprising since similar results were obtained in a previous publication in birds immunized with modified live and turkey herpesvirus (HVT)-IBDV vectored vaccines, where it was shown that immunity was able to delay but not totally prevent IBDV replication [39]. The overall variability of antibody titers, BF lesion scores and viral loads observed between chickens within the VP2-BP group may indicate that, in spite of antigen efficacy (demonstrated by survival to challenge), dose and/or schedule are not optimal. For this reason, future studies will be focused on defining immunization conditions (i.e. timing and dosage) that optimize efficacy.

IBD is still a serious problem for the poultry producers throughout the world. Most live traditional vaccines despite their high efficacy and widespread use, possess unstable antigenic and pathogenic characteristics, and can induce bursal atrophy [40]. Previous experiments with VP2-based vectored vaccines showed good protection against clinical disease but were unable to prevent development of lesions to the BF [39, 41, 42]. Immunization studies with VP2 produced in insect cells [43] or in yeasts [38] have shown good protection from clinical disease and bursal damage. Similarly, in our experiments, the immunization with the VP2-BP were able to induce not only a strong immune response towards vvIBDV but also protection from the onset of the disease and, in most animals, of the bursal lesions associated to viral infection. A recently published article comparing the efficacy in broilers of different commercially available new generation vaccines [44] showed that the best results, in terms of protection against challenge with vvIBDV, were obtained using the HVT-IBD vector vaccine (*Vaxxitek* ®) and the immune complex vaccine (*Bursa-Plex*®). Both these vaccines are based on live viruses and could present some environmental safety concerns. The antibody titers induced by the immunization with these vaccines were similar and sometimes lower compared to those induced by VP2-BP, such as bursal lesion scores.

In conclusion in this work, we demonstrate that by using a plant production strategy based on transient expression it is possible to obtain the formation of VP2-based supramolecular structures in plant cells and that these structures, rapidly purified through a simple sucrose cushion, when administered to chikens are able to activate immune responses conferring 100% of protection from challenge with vvIBDV, similar to what was obtained with the commercial inactivated IBD vaccine. The advantage of using the plant production platform is mainly in terms of costs, scalability, and safety [45]. Moreover, the His-pVP2 recombinant vaccine may allow the differentiation of infected from vaccinated animals (DIVA) through an appropriately designed diagnostic assay.

Further experiments will be needed to improve efficacy by testing different vaccination protocols and also to set- up an expression strategy of the recombinant antigen in edible plants that may allow mucosal delivery, paving the way to the development of an optimal IBDV vaccine.

## Supporting information

S1 Raw image (PDF)
