# Supplementary material for: The expression in plants of an engineered VP2 protein of Infectious Bursal Disease Virus induces formation of structurally heterogeneous particles that protect from a very virulent viral strain
Source: PLoS One. 2021 Feb 16;16(2):e0247134. doi: 10.1371/journal.pone.0247134 (PMC7886152; doi:10.1371/journal.pone.0247134)
Supplement: S1 Raw image — (PDF) [file pone.0247134.s001.pdf]

S1 Raw image

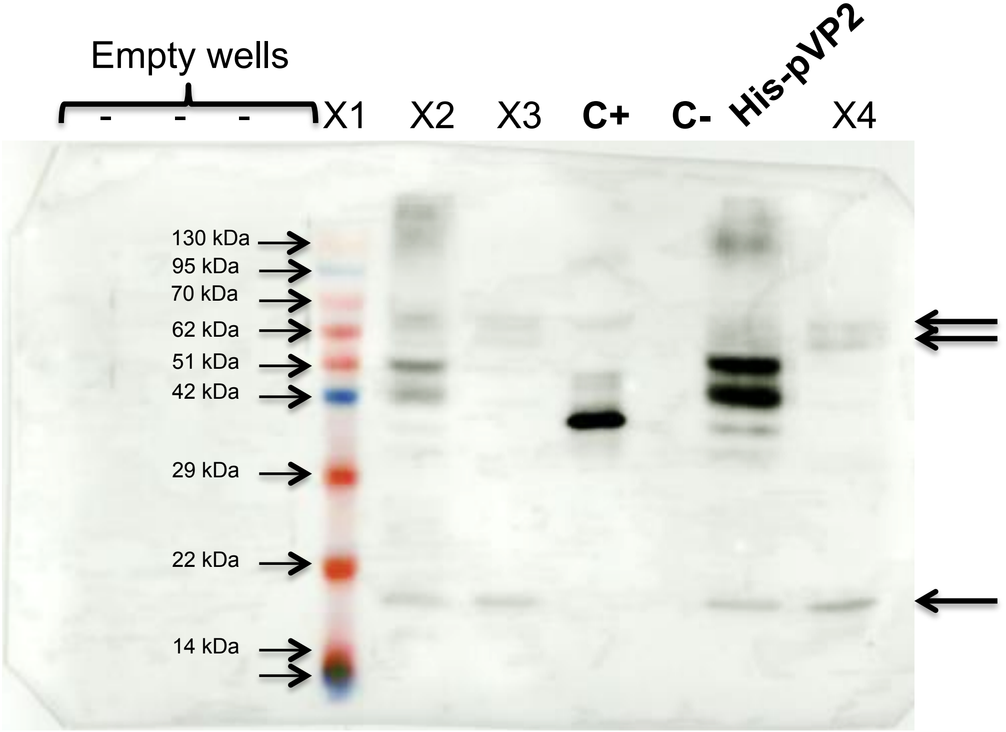

Western blot analysis of agroinfiltrated leaves expressing His-pVP2.
Plant extracts were separated by 11% SDS-PAGE, before electro-transfer to a polyvinylidene fluoride (PVDF) membrane (Millipore, Bedford, MA) using a Semi-Dry Transfer Unit (Hoefer TE70; GE Healthcare, Freiburg, Germany). As positive and negative controls, inactivated IBDV (provided by IZSVe) (C+) and the extract of plants infiltrated only with p35:AMCV-P19 (C-) were used respectively. The membranes were then blocked with 4% milk-PBS 2 h at room temperature, and after washing, the rabbit anti-VP2 serum diluted 1:1000 in 2% milk-PBS was added and incubated overnight at 4°C. After washings, the goat anti-rabbit HRP-conjugated antibody (ThermoFisher Scientific, Rockford USA) diluted 1:5000 in 2% milk-PBS for 1 h at 37 °C was added. Proteins were detected by enhanced chemiluminescence (ECL, Plus; GE Healthcare) using an ImageQuantTM LAS 500 system (GE Healthcare, Uppsala, Sweden).

Lanes marked with X not shown in Figure 1:

Lane X1: PiNK Plus Prestained Protein Ladder (GeneDireX, Inc.); Lane X2: 2 µg of His-pVP2 protein extract; Lane X3: 10 µg of P19 protein extract (C-); Lane X4: 10 µg of P19 protein extract (C-).

Lanes shown in Figure 1:

C+: 100 ng of inactivated IBDV; C-: 2 µg of P19 protein extract ; His-pVP2: 10 µg of His-pVP2 protein extract;

Faint bands indicated with an arrow were present both in the His-pVP2 and P19 (C-) protein extracts thus representing non-specific signals.
